# High-Efficiency Clustering Routing Protocol in AUV-Assisted Underwater Sensor Networks

**DOI:** 10.3390/s24206661

**Published:** 2024-10-16

**Authors:** Yuzhuo Shi, Xufeng Xue, Beibei Wang, Kun Hao, Haoyi Chai

**Affiliations:** 1 College of Information Technology, Tianjin College of Commerce, Tianjin 300350, China; 2 School of Computer and Information Engineering, Tianjin Chengjian University, Tianjin 300384, China; 3 School of Control and Mechanical Engineering, Tianjin Chengjian University, Tianjin 300384, China

**Keywords:** underwater sensor networks, remaining energy, node degree, AUV, dynamic relay node

## Abstract

Currently, underwater sensor networks are extensively applied for environmental monitoring, disaster prediction, etc. Nevertheless, owing to the complicacy of the underwater environment, the limited energy of underwater sensor nodes, and the high latency of hydroacoustic channels, the energy-efficient operation of underwater sensor networks has become an important challenge. In this paper, a high-efficiency clustering routing protocol in AUV-assisted underwater sensor networks (HECRA) is proposed to address the energy limitations and low data transmission reliability in underwater sensor networks. The protocol optimizes the cluster head selection strategy of the traditional low-energy adaptive clustering hierarchy (LEACH) protocol by introducing the residual energy and node degree in the cluster head selection phase and performs some optimizations in the cluster formation and data transmission phases, including selecting clusters for joining by ordinary nodes based on the residual energy of the cluster head nodes and weight computation based on the depth and residual energy of the cluster head nodes to select the optimal message forwarding nodes. In addition, this paper introduces an autonomous underwater vehicle (AUV) as a dynamic relay node to improve network transmission efficiency. According to the simulation results, compared with the existing LEACH, the energy efficient routing protocol based on layers and unequal clusters in underwater wireless sensor networks (EERBLC) and energy-efficient clustering multi-hop routing protocol in a UWSN (EECMR), the HECRA significantly improves network lifetime, the residual node energy, and the number of successfully transmitted packets, which can effectively prolong network lifetime and ensure efficient data transmission.

## 1. Introduction

Currently, underwater sensor networks have become a crucial and challenging technology in scientific research and practical applications and have been widely used in acquiring underwater data and disaster warnings [1,2,3]. Nevertheless, owing to the special properties of underwater environments, such as high pressure, high salinity, temperature fluctuations, and current mobility, numerous challenges exist in the design and operation of underwater sensor networks, such as energy limitations, hydroacoustic signal attenuation, high transmission delays, and decreased reliability of data transmission [4,5]. Apart from that, the energy of nodes is limited, and it is very complicated to replace batteries [6]. Therefore, reducing node energy consumption and improving network energy efficiency have become important issues in the development of underwater sensor network technology [7].

Traditional clustering protocols are effective in achieving energy efficiency in underwater sensor networks. LEACH [8] is a classical clustering protocol that organizes the network nodes into multiple clusters by rotating the cluster head nodes to reduce node energy consumption. Nevertheless, LEACH has several limitations when dealing with underwater sensor networks, such as the inability to adapt to the complicacy and variability of the underwater environment, and the random cluster head selection method may lead to an unbalanced load on some cluster head nodes, which accelerates energy consumption and leads to premature energy depletion at some nodes, thus affecting the network lifetime and reliability. In recent years, many scholars have conducted extensive research on clustering protocols for underwater sensor networks. Li et al. [9] introduced a Controlled-LEACH protocol (C-LEACH), which involves the deployment of a control node at the central position of the network topology; the control node handles operations such as cluster head selection and cluster formation, which greatly reduces the overhead of the sensor nodes in the network and effectively extends the network lifetime. Nevertheless, the processing time of the control node is longer, which increases the network latency. Xu et al. [10] introduced an improved revised cluster routing algorithm named E-LEACH, which places nodes with higher residual energy as cluster head nodes. In the first round, all the nodes have equal chances of being selected as cluster head nodes; after the second round, the residual node energy starts changing, and the cluster head node is taken as the node with the highest residual energy. Compared to LEACH, this protocol improves selection, which saves energy and effectively extends the network lifetime but ignores the network connectivity problem. Gomathi et al. [11] proposed an improved routing strategy named the EE-MDCHSRP, which dynamically selects the cluster head nodes by considering the node density, the residual node energy, and the minimum migration factor. This approach can effectively balance the cluster head node’s energy and extend the network lifetime, but frequent cluster head election leads to considerable unnecessary energy consumption. Hong et al. [12] proposed a new protocol named TCEB, which takes energy and path loss as influencing factors in selecting cluster heads and represents the topology control protocol as a noncooperative game, with a payoff function consisting of residual node energy and one-hop distance path loss, which can effectively distribute the energy consumption among all nodes. However, this protocol does not consider the mobility of sensor nodes. Nikolidakis et al. [13] introduced a routing protocol called ECHERP, which focuses on balanced cluster head election. This protocol utilizes a balanced clustering approach and Gaussian elimination algorithm to choose a random node or a node with higher residual energy as a new cluster head at a specified time. The protocol also plans the number of cluster head cycles based on residual energy in order to prolong the network’s lifetime. Nevertheless, this protocol does not consider the network’s transmission efficiency. Khan et al. [14] proposed an improved routing protocol that uses the AUV for cluster head selection, cluster formation, data transmission, and sleep–wake scheduling, which greatly saves the sensor node energy; nevertheless, cluster head nodes must wait for the AUV to collect the data, which increases the network latency. Zhu et al. [15] presented a routing protocol called EERBLC that focuses on enhancing energy efficiency in routing by utilizing layers and unequal clusters, which proposes a new cluster head selection method based on energy balance and node degree and a forwarding node calculation method based on the forwarding rate and residual energy. This approach can prolong the network’s operational lifetime and enhance data transmission capabilities, but the protocol lacks consideration of node mobility. Nguyen et al. [16] proposed the EECMR, which divides the network into layers and selects the cluster heads based on the residual energy and node depth, which can effectively prolong the network lifetime; Nevertheless, the communication before the layers is too dependent on relay nodes, and the communication latency is high.

To summarize, some scholars have also failed to fully consider the residual energy of nodes, connectivity of nodes, and data transmission efficiency in their studies, and the application of AUV is more limited in previous studies. Based on this, HECRA is proposed in this paper. The innovations and contributions made in this paper are as follows:

(1) The protocol introduces node residual energy and node degree as influencing factors in the cluster head selection phase, which makes the energy of nodes more balanced.
(2) The protocol formulates a communication strategy based on the depth and residual energy of the cluster head nodes in the data transmission phase to optimize the message forwarding path.
(3) In this paper, we innovatively introduce AUV as a dynamic relay node for transmitting messages between cluster-head nodes that are unable to directly communicate with each other, which improves the transmission efficiency and stability of the network.

The rest of the paper is structured as follows. The proposed system model is covered in Section 2. The detailed design process for HECRA is discussed in Section 3. Section 4 evaluates the performance of the proposed HECRA and compares it with existing cluster routing algorithms. Finally, Section 5 concludes the paper.

## 2. System Model

**Figure 1:**
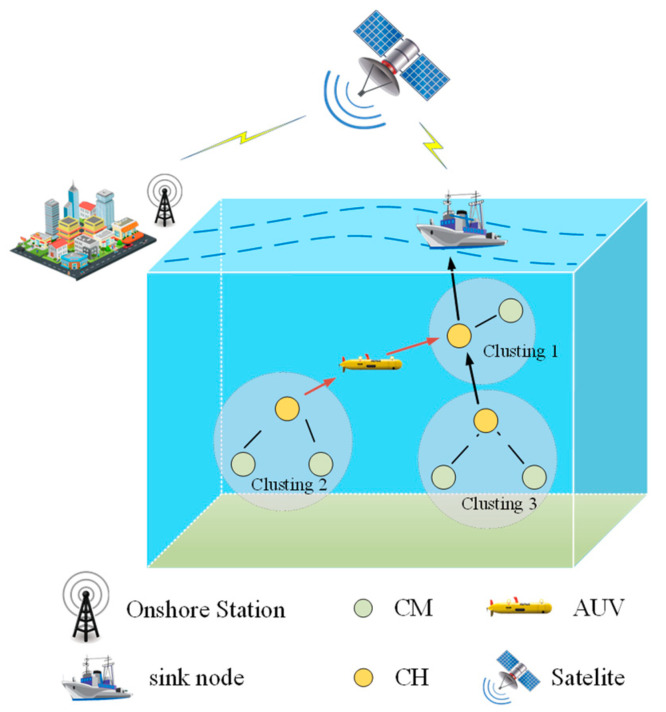
Network model.

The underwater sensor network consists of sink nodes, sensor nodes, and an AUV. The sink nodes are deployed on the water surface, and after satellite relaying, they can transmit data to and from the ground base station via radio communication. Each sensor node may become a cluster head (CH) node or a cluster member (CM) node. In each round of communication, the CHs fuse the data from the CMs and send the information to the sink node through one-hop or multihop communication, while the AUV acts as a dynamic relay node for relaying messages between CHs that cannot communicate owing to long distances. The network model is shown in Figure 1.

Within this study, the energy consumption model for hydroacoustic communication described in the literature [17] is used to describe the energy scenario of the network, assuming that a sensor node transmits kb bits of data at a transmission rate $T_{r}$ over a distance d. The energy consumed by the sensor node is defined as follows:

(1) $$E_{T}=\frac{kb}{T_{r}}*P_{tx}$$

where $P_{tx}$ is the transmission power of the node.

If a sensor node receives kb bits of data, the energy consumed by that sensor node is defined as follows:

(2) $$E_{R}=\frac{kb}{T_{r}}*P_{rx}$$

where $P_{rx}$ is the received power of the node.

It is also important to consider the data fusion energy consumption, which is the amount of energy required to fuse kb bits of data:

(3) $$E_{A}=kb*E_{DA}$$

where $E_{DA}$ is the energy consumed to fuse the data per unit length.

In underwater sensor networks, the movement of nodes with ocean currents is a crucial factor. In this paper, we use the meandering current mobility model (MCM) [18] to simulate node movement. The MCM uses a two-dimensional model to simulate the movement of underwater nodes because oceans are rotating stratified fluids, and most of the vertical motion can be ignored. Assuming that the coordinates of the plane of the node with deployment number i are $\left(x_{i},y_{i}\right)$, the movement function of this node in time $t_{m}$ can be given by the following equation:

(4) $$\Psi \left(x_{i},y_{i},t_{m}\right)=-\tanh\left[\frac{y_{i}-B\left(t_{m}\right)\sin\left(k\left(x_{i}-ct_{m}\right)\right)}{\sqrt{1+k^{2}B^{2}\left(t_{m}\right)\cos^{2}\left(k\left(x_{i}-ct_{m}\right)\right)}}\right]$$

where k is the number of meanders in the unit length, which is usually 2π/7.5. c is the phase speed at which the phases shift downstream, usually 0.12. 

$B(t_{m})$ is the current amplitude function, defined as follows:

(5) $$B\left(t_{m}\right)=A+\varepsilon \cos\left(\omega t_{m}\right)$$

where A  denotes the average width of the ocean current; usually 1.2. ϵ is the modulation amplitude, usually 0.3. ω is the modulation frequency, which can be taken as 0.4.

Therefore, the plane coordinates $\left({\dot{x}}_{i},{\dot{y}}_{i}\right)$ of sensor node i  after moving with the ocean current after time $t_{m}$ can be calculated by the following equation:

(6) $${\dot{x}}_{i}=-\frac{\partial \Psi \left(x_{i},y_{i},t_{m}\right)}{\partial y_{i}}$$

(7) $${\dot{y}}_{i}=\frac{\partial \Psi \left(x_{i},y_{i},t_{m}\right)}{\partial x_{i}}$$

## 3. Design of the HECRA

Conventional LEACH methods have several drawbacks in terms of energy efficiency and data transmission reliability, which are more significant, especially in underwater environments. The random CH selection method of LEACH may lead to the overloading of some CHs, which accelerates energy consumption. In addition, hydroacoustic signals are more susceptible to interference and attenuation, threatening the reliability of data transmission.

Given the analysis above, this paper proposes HECRA, which aims to optimize the performance of underwater sensor networks. HECRA employs more energy-efficient cluster head selection strategies and data transmission strategies to balance the node loads and prolong the network lifetime. HECRA is divided into four phases: cluster head selection, cluster formation, data transmission, and network optimization, which are described in detail below.

### 3.1. Cluster Head Selection Phase

In the initial state, after the node deployment is completed, each node has no information about the surrounding environment; at this time, each node broadcasts a “HELLO” message or the message content node ID information. When the node successfully receives the “HELLO” message, the sender’s ID is input to the cache, and its node degree is increased by 1. The node’s waiting time is set to the node with the maximum distance or transmission propagation delay.

By calculating its threshold function, each node determines whether to act as a CH during the present round. This paper introduces the residual node energy and node degree influence factor on the basis of traditional LEACH, mainly considering the residual energy E, and node degree $Ne_{i}$ of node i. This is due to the consideration of node residual energy, which can prioritize the nodes with higher residual energy to act as cluster heads, thus avoiding the low-energy nodes from exhausting their energy prematurely, resulting in a shorter network lifetime. Considering the node degree also ensures that the elected cluster head nodes have better connectivity and can communicate better with the surrounding nodes. The improved threshold function $T_{i}^{’}$ is defined as follows:

(8) $$\left\{\begin{array}{l} T_{i}^{’}=\frac{F}{\max\_F}\\ F=\frac{p}{1-p\left(rmod\frac{1}{p}\right)}+\lambda_{1}\frac{E_{i}}{E_{Init}}+\lambda_{2}\frac{Ne_{i}}{N} \end{array}\right.$$

where r represents the number of network operation rounds, p indicates the percentage of expected CHs to the total number of network nodes in the current round, $E_{init}$ is the initial node energy, N  is the total number of nodes, $\lambda_{1}$ and $\lambda_{2}$ are the weighting coefficients, which are used to regulate the weight of the residual energy and the node degree, and $\lambda_{1}+\lambda_{2}=1$ and max⁡_F are the maximum values of F.

Then, node i  generates a random number rand  that is greater than 0 and less than 1 and determines whether it is a CH by comparing rand  with the improved threshold function $T_{i}^{’}$. By this calculation method, nodes with greater residual energy and higher degrees are more prone to being chosen as CHs, which can solve the problem of early death of CHs owing to rapid energy depletion and can also improve network communication efficiency, thus effectively prolonging the lifespan of the network.

### 3.2. Cluster Formation Phase

Once the process of cluster head selection is finished, the CH broadcasts the cluster formation message to its communication range. Each CM node listens to its surroundings during this phase to receive cluster formation messages from different nodes. These messages contain the ID and residual energy information of the corresponding CH.

Depending on the received cluster formation messages, the sensor node will select clusters based on the following. If the sensor node receives only one cluster formation message, it will join the corresponding CH. If the sensor node receives more than one cluster formation message, it evaluates these messages and joins the cluster where the CH with more residual energy is located. In particular, because the CHs $CH=\{CH_{1},CH_{2},\ldots ,CH_{m}\}$ corresponding to the m received cluster formation messages, the sensor node j  will select its own CH $H_{j}$ based on the following formula:

(9) $$H_{j}=CH_{\underset{i}{\arg max}\left(E_{i}\right)},1\leq i\leq m$$

where i is the number of sending message CHs, $E_{i}$ denotes the residual energy of the sending message CH, and node j selects the CH with the maximum residual energy and joins the cluster.

If the sensor node does not receive the cluster formation message, it proves that the node cannot communicate with any other node; then, the node goes into a sleeping state and waits for the broadcast message.

### 3.3. Data Transmission Phase

After the cluster formation, the CH sends a control message, which causes the CMs to enter a sleeping state, sense the environment, and collect the data. During the intracluster communication phase, the CH sends a wake-up message, which is received by the CMs, and sends the collected data to the CH, which conducts operations such as fusion.

**Figure 2:**
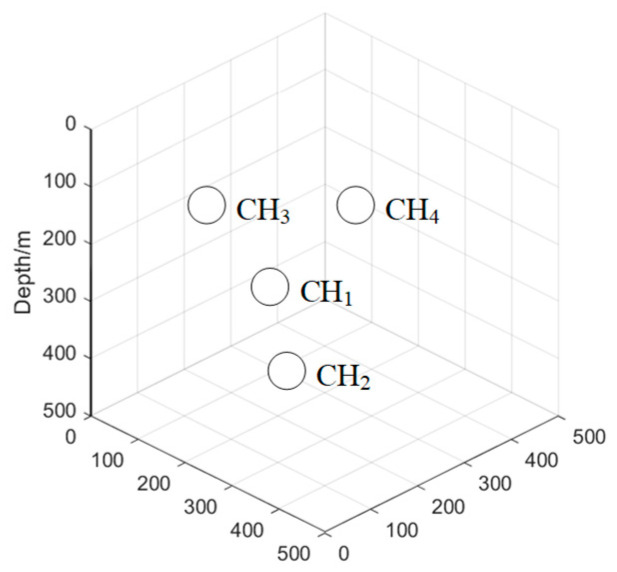
CH pathfinding schematic.

In the intercluster communication phase, the communication between CHs involves multihop transmission; specifically, the CH broadcasts a pathfinding message, including its node ID and depth information; if another CH receives a pathfinding message, the CH that receives the message determines whether its own depth is shallower than that of the node that sent the message, replies with a confirmation message if it meets the condition, and if it does not meet the condition, it ignores the message and replies with a confirmation message after it receives a pathfinding message sent from the same node for the second time. As shown in Figure 2, the $CH_{1}$ broadcasts a pathfinding message within its transmission distance, $CH_{2}$, $CH_{3}$, and $CH_{4}$ receive the pathfinding message because the depth of $CH_{2}$ is greater than $CH_{1}$; thus, it does not reply to node $CH_{1}$, while $CH_{3}$ and $CH_{4}$ are all smaller than $CH_{1}$; thus, $CH_{3}$ and $CH_{4}$ reply to the $CH_{1}$ confirmation message, and the content of the message includes its node ID and the forwarding weights. The formula for the forwarding weights is as follows:

(10) $$\omega_{i}=\alpha \frac{E_{i}}{E_{Init}}+\beta \left(1-\frac{D_{i}}{\max\_D}\right)$$

where i  is the number of current CHs, $E_{i}$ is the residual energy of the node, $E_{init}\;$ is the initial energy of the node, $D_{i}$ is the depth of the node, max⁡_D is the maximum depth of the ocean, and α and β are weighting coefficients that are used to balance the effects of two factors, namely, energy and depth, respectively, and α+β=1.

After the $CH_{1}$ receives the confirmation message replied to by node $CH_{3}$ and node $CH_{4}$, it compares the forwarding weights of $CH_{3}$ and $CH_{4}$ and selects the CH with the largest weight as the message forwarding node.

### 3.4. Network Optimization Phase

**Figure 3:**
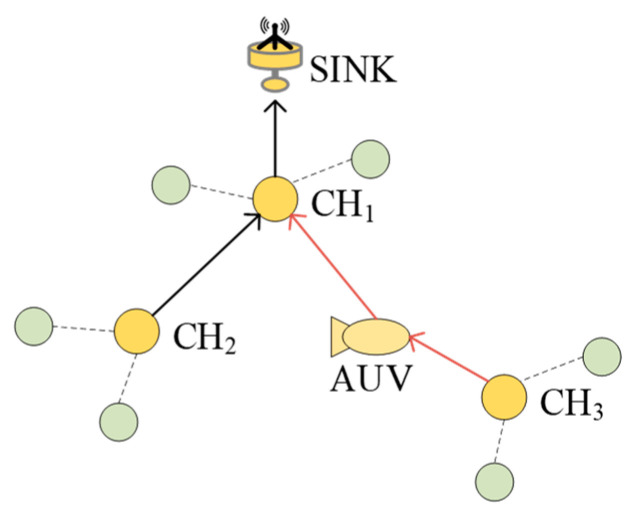
AUV dynamic relay schematic.

Owing to the limited communication distance between CHs, there may be situations where some CHs cannot communicate directly. For example, in Figure 3, $CH_{3}$ and $CH_{1}$ cannot communicate directly owing to the long distance, and this situation will greatly reduce the network transmission efficiency. To solve this problem, this paper introduces AUV assistance, which allows the AUV to act as a dynamic relay node to relay messages from CHNs, thus improving transmission efficiency and network performance.

The AUV follows a predefined path and determines clusterhead locations and distances in the underwater sensor network using existing localization methods. Based on the detected network topology information, the $CH_{3}$ that cannot communicate directly with other CHs is identified, and the AUV calculates the closest $CH_{1}$ that can communicate with the sink to $CH_{3}$ and then moves to the midpoint position of $CH_{1}$ and $CH_{3}$ to act as a dynamic relay node and forward the message from $CH_{3}$ to $CH_{1}$. After completing the transmission, the AUV continues to the next relay position to perform the task. The pseudocode for the specific steps of HECRA is as follows (Algorithm 1).


**Algorithm 1. HECRA**
**Input:** node ID, initial energy, depth**Output:** Cluster results
**State 1: Network Initialization**
    **For** each node *i*     Broadcast HELLO = {node ID}      **If** receive HELLO       
$Ne_{i}=Ne_{i}+1$      **End If**    **End For**
**State 2: Cluster head selection and cluster formation**
    **For** each node *i*     Calculate weight
$T_{i}^{’}$ as in (9)     Generate a random number
0≤rand≤1      **If **
$rand<T_{i}^{’}$       Elect node *i* to become CH      **Else if** receive Cluster formation message       Calculate weight
$H_{j}$ as in (10)       Join the cluster corresponding to the CH
$H_{j}$      **Else**       Node goes to sleep      **End If**    **End For**
**State 3: Data transmission**
    **For** all CHs     Collecting and fusing information from nodes in the cluster     Broadcast wayfinding message      **If** receive wayfinding message       Determines whether to reply to the sender of wayfinding message      **End If**      **If** receive messages of confirmation       Calculate weight
$\omega_{i}$ as in (11)       Find the node that forwarded the message      **End If**    **End For**
**State 4: Optimization of the network**
   AUV seeks CHs that are unable to communicate with other nodes   Find the location of the relay transmission   Assisted in completing data forwarding and continued cruising

## 4. Simulation and Performance Evaluation

### 4.1. Simulation Environment

In this paper, the simulation is performed on the MATLAB platform. The underwater environment area is set to 500 × 500 × 500 m, the quantity of sensor nodes varies between 50 and 450, and the transmission distance of each node is set to 150 m or 200 m [16]. The sink node is positioned on the sea surface, situated at the central right area of the sea surface. Each sensor node sends two data packets. Table 1 presents the values of additional parameters utilized in the simulation.

**Figure 4:**
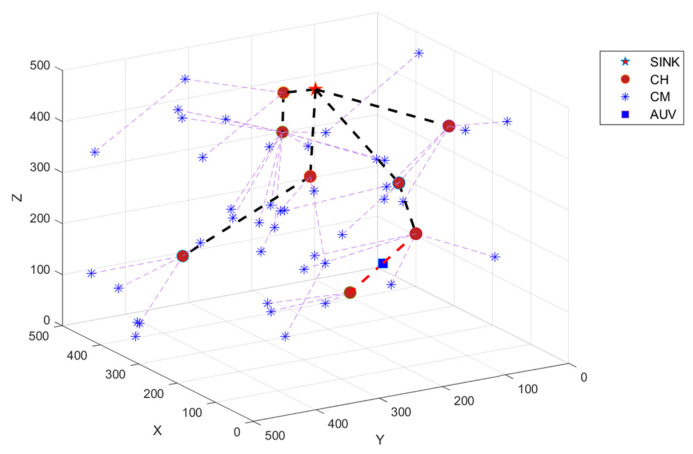
Network after HECRA.

Figure 4 shows the network after implementing the HECRA under a 500 × 500 × 500 m area with 50 sensor nodes and a transmission distance of 200 m. The figure includes the sink, the CH, the CM, and the AUV that acts as a dynamic relay between the CHNs. The light blue dashed lines indicate intracluster communication, the black dashed lines indicate intercluster communication, and the red dashed lines indicate AUV relay communication.

### 4.2. Simulation Results

#### 4.2.1. Network Lifetime

The network lifetime is generally defined as the total time that all nodes survive [19]. When the residual energy of a node is 0, the node dies; this paper measures the network lifetime in terms of the number of deaths of the nodes. To evaluate the network lifetime of HECRA in different network environments, experiments are carried out with different numbers of nodes and different transmission range scenarios.

**Figure 5:**
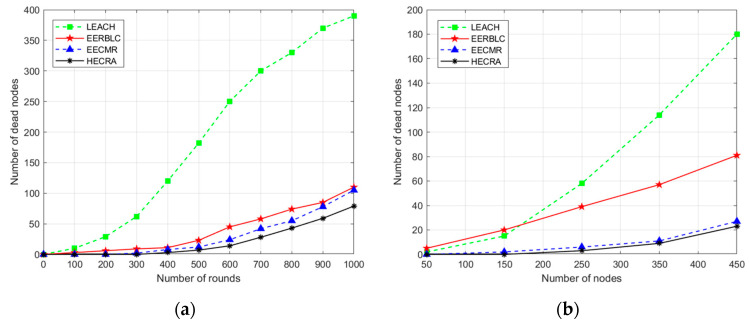
Number of dead nodes. (**a**) Transmission range 200 m, 450 nodes in 1000 rounds. (**b**) Transmission range 150 m, nodes 50 to 450.

Figure 5a shows the number of deaths of nodes for 1000 rounds of network operation when the transmission range is 200 m, and the number of sensor nodes is 450. The graphical results show that LEACH has an excessive number of dead nodes owing to unbalanced node energy consumption caused by the randomness of CH node selection, whereas the EERBLC, EECMR, and HECRA node deaths are slower overall because all three protocols are optimized for cluster head selection. HECRA further reduces the number of node deaths by implementing a node sleep–wake strategy. After 1000 rounds of network operation, HECRA reduces the number of dead nodes by 1.88%, 3.86%, and 36.57% compared to that of EECMR, EERBLC, and LEACH, respectively.

Figure 5b shows the number of dead nodes for a transmission range of 150 m and a varying number of sensor nodes between 50 and 450, where the number of sensor nodes is varied every 1000 rounds of operation. The graphical results show that the average number of dead nodes in HECRA is reduced by 0.8%, 12.87%, and 20.18% compared to that in EECMR, EERBLC, and LEACH, respectively, for varying numbers of nodes, where LEACH performs the worst in terms of the number of node deaths because all the nodes are randomized to become CHs sequentially in this protocol, which leads to unbalanced energy consumption. The EERBLC achieves more balanced energy consumption by optimizing the cluster head selection considering the node degree and residual energy; hence, the number of node deaths is lower than that of the LEACH algorithm. EECMR stratifies the network based on depth on the basis of CH selection and introduces relay nodes to reduce energy consumption further; hence, the number of node deaths is lower. HECRA is optimized for the CH data at the data transmission stage for multihop transmission, and dynamic relay nodes are introduced to save energy more effectively; hence, the number of node deaths is slightly lower than that of EECMR.

#### 4.2.2. Residual Energy of Nodes

To evaluate the residual energy of the HECRA nodes in different network environments, experiments are conducted in this paper with different numbers of nodes and different transmission ranges.

**Figure 6:**
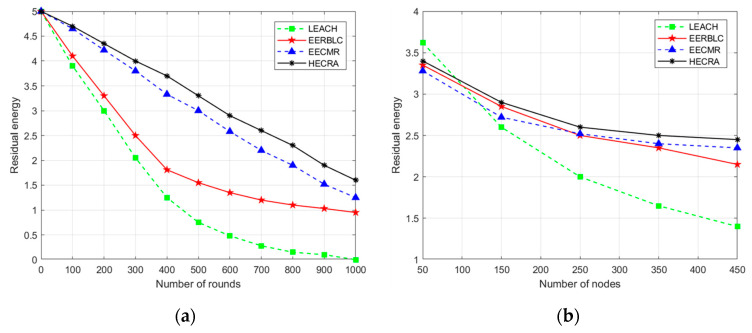
Average residual energy of a node. (**a**) Transmission range of 200 m, 450 nodes in 1000 rounds. (**b**) Transmission range of 150 m, nodes 50 to 450.

Figure 6a shows the residual energy of the nodes for 1000 rounds of network operation when the transmission range is 200 m and the number of sensor nodes is 450. The graphical results show that the residual node energy of LEACH decreases the fastest when the network runs, and the residual node energy of HECRA improves by 5.27%, 22.65%, and 35.25% compared to that of EECMR, EERBLC, and LEACH, respectively.

Figure 6b shows the residual node energy for the case where the transmission range is 150 m and the number of sensor nodes varies between 50 and 450 nodes, where the number of sensor nodes is varied every 1000 rounds of operation. The graphical results show that the residual energy of LEACH is greater than that of the other protocols when the number of nodes is less than 100 because, in sparse networks, LEACH has a lower success rate of network communication when the transmission range is small, which results in less node energy consumption. Nevertheless, as the number of nodes increases, the energy consumption of LEACH gradually increases. Compared to the other three protocols, HECRA maintains better performance in both sparse and dense regions of nodes. This is because HECRA optimizes the cluster head selection, data transmission, and dynamic relay nodes. HECRA maintains relatively good node residual energy in both node-sparse and -dense regions.

#### 4.2.3. Number of Successful Packet Transmissions

To evaluate the packet transmission of HECRA in different network environments, experiments involving different numbers of nodes and transmission ranges are conducted in this paper. In addition to the network lifetime and residual energy, the number of successful packet transmissions is also an important indicator of network performance. An efficient network needs to save energy while maintaining a high data transmission success rate.

**Figure 7:**
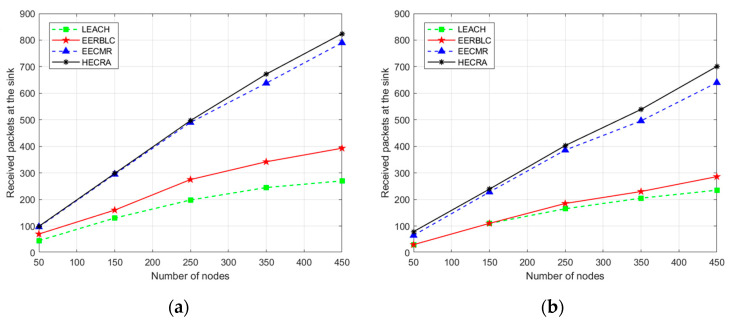
Received packets at the sink. (**a**) Transmission range 200 m, nodes 50 to 450. (**b**) Transmission range 150 m, nodes 50 to 450.

Figure 7a,b show the number of successful packet transmissions when the transmission range is 200 m and 150 m, respectively, with the number of sensor nodes varying between 50 and 450 and the number of sensor nodes varying every 1000 rounds of operation. From the graphical results, it can be seen that when the transmission range decreases, the number of successful packet transmissions for the four protocols also decreases, while HECRA still shows better performance overall; as the number of nodes varies, the number of successful packet transmissions for a transmission range of 200 m compared to EECMR, EERBLC, and LEACH is improved by 2.61%, 42.89%, and 58.47%, respectively. The number of successful packet transmissions for a transmission range of 150 m is improved by 6.8%, 45.17%, and 47.82% compared to that of EECMR, EERBLC, and LEACH, respectively. This is mainly because HECRA introduces an AUV as a dynamic relay node, which enables it to effectively assist in data transmission when the CH is unable to communicate. As a result, HECRA significantly improves the success rate of packet transmission and enhances the throughput and stability of the network.

## 5. Conclusions

In this work, a high-efficiency clustering routing protocol is proposed for AUV-assisted underwater sensor networks. In this paper, the computation of the threshold value of the cluster head selection phase is improved based on the traditional LEACH algorithm by introducing the residual node energy and the node degree influence factor to adjust the probability of cluster head node selection. Sensor nodes are selected to join the cluster based on the residual energy of the CH. In addition, this paper also improves the intercluster communication strategy based on the residual energy and depth of the CH. This paper introduces an AUV as a dynamic relay node to improve network stability. By analyzing the simulation results, this paper finds that HECRA exhibits a longer network lifetime, higher residual energy of nodes, and a higher packet transmission success rate in different network environments, which indicates that the improvements in cluster head selection and data transmission in this paper effectively improve the network lifetime and throughput rate. Nevertheless, in the next step, this paper needs to address the issue of rational path planning for AUVs as relay nodes to better improve network efficiency through better transit operation routes.

## Figures and Tables

**Table 1:** Simulation parameters.

Simulation Parameters	Value
Initial node energy	5 J
Data packet size	200 bits
Transmit power	2 W
Receive power	0.1 W
Number of sink nodes	1
Acoustic frequency	30 kHz
Rounds	1000

## Data Availability

The data used to support the findings of the study can be obtained from the corresponding author upon request.
